# Soluble P-selectin promotes retinal ganglion cell survival through activation of Nrf2 signaling after ischemia injury

**DOI:** 10.1038/cddis.2017.566

**Published:** 2017-11-16

**Authors:** Kishan Kapupara, Yao-Tseng Wen, Rong-Kung Tsai, Shun-Ping Huang

**Affiliations:** 1 Department of Molecular Biology and Human Genetics, Tzu Chi University, Hualien, Taiwan; 2 Institute of Eye Research, Buddhist Tzu Chi General Hospital, Hualien, Taiwan; 3 Institute of Medical Sciences, Tzu Chi University, Hualien, Taiwan

## Abstract

Retinal ischemic injuries play an important role in the pathogenesis of several eye disorders. Inflammation and oxidative stress are key players in ischemic injuries. Following retinal ischemia, vascular endothelial cells and leukocytes express several inflammatory adhesion receptors, such as selectins and cell adhesion molecules. P-selectin stimulates leukocyte recruitment to platelet aggregates and has an important role in vascular homeostasis and inflammatory leukocyte extravasation. Soluble P-selectin can be neuroprotective through competitive binding to the receptors of endogenous P-selectin molecules. Here, we demonstrate the neuroprotective effect of a recombinant P-selectin immunoglobin G (P-sel-IgG) chimeric fusion protein in a rat anterior ischemic optic neuropathy (rAION) model. rAION was induced by photodynamic therapy. P-sel-IgG treatment reduced optic nerve edema and stabilized the blood–optic nerve barrier (BONB) in the acute phase of rAION. Further, P-sel-IgG increased the retinal ganglion cell (RGC) survival rate, reduced RGC apoptosis, preserved visual function, maintained retinal nerve fiber layer thickness, and reduced macrophage infiltration in optic nerve tissue in the chronic phase (day 28). Increased NAD(P)H quinone dehydrogenase 1 (NQO1) and heme oxygenase 1(HO-1) expression levels, along with increased transcription factor Nrf2, suggesting an antioxidant role of P-sel-IgG via the Nrf2 signaling pathway. In conclusion, this study is the first to demonstrate that P-sel-IgG treatment promotes RGC survival by stabilizing the BONB and activating the Nrf2 signaling pathway in a rAION model.

Retinal ischemia, which leads to profound vision loss, is a common pathology in many eye disorders, including ischemic optic neuropathies,^1^ diabetic retinopathy,^2^ retinal artery occculsion,^3^ choroidal neovascularization (CNV)^4^ and glaucoma.^5^ Retinal ischemia involves reduced oxygen, metabolites and waste product clearance. Damage to the retina, an extension of the central nervous system (CNS), is irreversible and can result in the death of retinal ganglion cells (RGCs),^6^ amacrine cells,^7^ and bipolar cells,^2^ depending on the disease type and status. Retinal ischemia induced-optic disc drusen (crowded optic nerve),^8, 9^ impaired retinal vasculature,^10^ hemorhage,^11^ neovascularization,^4^ and retinal detachment^2^ cause vision loss. The pathophysiology aspects of retinal ischemic diseases have been studied previously and various mechanisms have been hypothesized. Disease mechanisms that may lead to cell death are oxidative stress in the retina,^1^ expression of pro-inflammatory factors in the optic nerve,^12^ disruption of calcium ion homoeostasis,^13^ and macrophage polarization.^12^ Considering these mechanisms, some strategies can reduce tissue damage with anti-inflammatory compounds,^14^ neurotropic factors,^4, 12^ oxidative stress regulators,^15, 16^ calcium channel blockers^17^ and microglial activation inhibitors or blood-borne macrophage infiltration blockers.^18^ The rat anterior ischemic optic neuropathy (rAION) model represents an excellent model to investigate RGC pathology and ischemic injury because rAION shares similar features and pathology with human and primate AION.^1^

The rAION model used in this study is achieved by photodynamic therapy, which generates superoxide radicals that circulate within optic nerve (ON) capillaries,^19, 20^ causing ON infarct and ischemia. Inflammation and oxidative stress generated by reactive oxygen species (ROS) in rAION cause RGC death. Therefore, reducing this inflammatory response and oxidative stress can prevent RGC apoptosis.

P-selectin (CD62), a member of the selectin family, is confined to the *α*-granules of platelets and Weibel-Palade bodies of endothelial cells.^21^ P-selectin is translocated to the surface upon activation of endothelial cells^22^ or platelets^23^ for leukocyte recruitment. The P-selectin–PSGL-1 (P-selectin glycoprotein ligand-1) interaction supports leukocyte rolling and firm adhesion, leading to transmigration in surrounding tissue that triggers an inflammatory response cascade.^24^ A soluble recombinant form of exogenous P-selectin can restore hemostasis in a mouse model of hemophilia,^25^ rescue viper venom-induced mortality,^26^ rescue liver endothelial cells from ischemic reperfusion injury^27^ and ameliorate inflammation.^28^ All these findings are based on one common principle; the soluble recombinant form of exogenous P-selectin competes with endogenous membrane bound P-selectin molecules to bind with PSGL-1, a well-known ligand for P-selectin.^26, 27^ Considering the similar pathophysiology in rAION, including ischemia, photothrombosis,^1^ and inflammation,^29^ we investigated the therapeutic potential of soluble P-selectin in ischemic injury. We hypothesize that P-sel-IgG treatment can be neuroprotective for the ON and RGCs, resulting in preserved visual function. In addition, stopping the inflammatory process is a potential therapeutic target, but little is known about the antioxidative pathway in rAION.

Oxidative stress caused by the production of ROS triggers a stress response via the nuclear factor erythroid 2-related factor 2 (Nrf2)-antioxidant response element (ARE) signaling axis, which scavenges ROS and maintains redox status.^30^ It was thought that Nrf2 was limited to redox control and that anti-inflammatory effects were the result of the elimination of ROS by Nrf2. However, Nrf2 inhibits the transcription of pro-inflammatory cytokines by binding in close proximity to these genes in ARE-dependent manner.^31^ Therefore, we are interested in further exploring the antioxidant pathway as an inflammatory counterpart in rAION, which has been shown in our previous report^12^ and that of another group.^29^

Assuming that P-sel-IgG will bind to PSGL-1, the present study examines the mechanism by which P-sel-IgG affects visual function, RGC survival, the blood–optic nerve barrier (BONB) and leukocyte recruitment after ischemic injury.

## Results

### P-sel-IgG treatment preserves visual function

**Figure 1:**
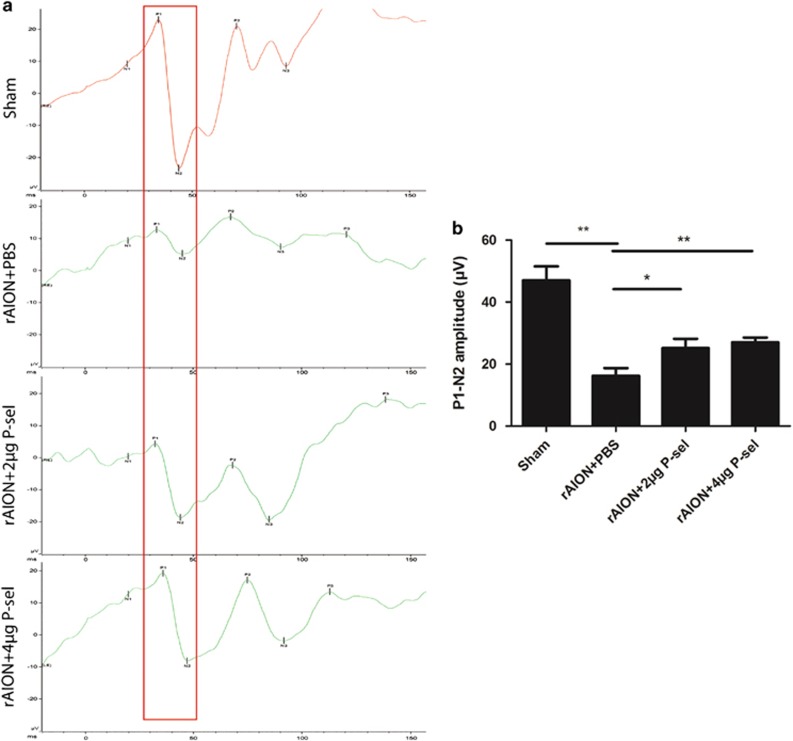
FVEPs. (**a**) Representative FVEP profile after rAION in each group (the red box indicates the P1-N2 amplitude). (**b**) Bar charts showing the P1-N2 amplitude. The amplitudes of the 4 *μ*g P-sel- and 2 *μ*g P-sel-treated groups were significantly higher than those of the PBS-treated group (25.16571±7.931084 *μ*V and 16.296±5.484773 *μ*V, respectively). Data are expressed as the mean±S.D.; *P⩽0.05, **P⩽0.01; *n*=6

Flash visually evoked potentials (FVEPs) were measured at day 28 post-infarct. The P1-N2 amplitudes in the sham, PBS-, 2 *μ*g P-sel- and 4 *μ*g P-sel-treated groups were 47.00±10.15, 16.29±5.5, 25.16±7.9 and 27.02±3.4 *μ*V, respectively. The P1-N2 amplitude was significantly preserved (Figure 1; 2 *μ*g P-sel, *P*=0.05; 4 *μ*g P-sel, *P*=0.008) in both treatment groups. These data suggest that P-sel-IgG can preserve visual function in the rAION model.

### P-sel-IgG treatment increases the RGC survival rate

**Figure 2:**
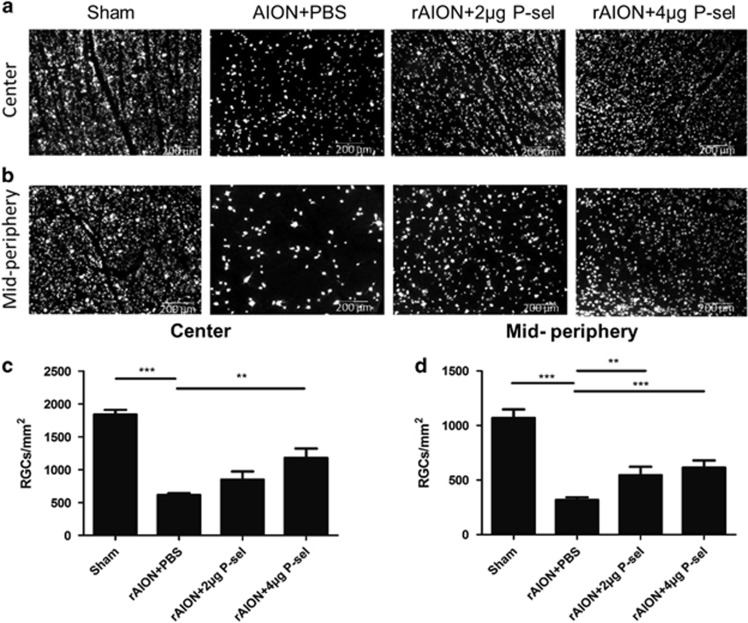
Retinal flat mount preparations and RGC morphometry. (**a**,**b**) Representative image of RGC density after rAION in each group. The 4 *μ*g P-sel-treated group showed significantly higher RGC density than the PBS-treated group in the (**c**) central (1009±177/mm^2^ *versus* 612±31/mm^2^, respectively) and (**d**) mid-peripheral retina (614±99/mm^2^ *versus* 323±92/mm^2^, respectively). The 2 *μ*g P-sel-treated group also showed significantly higher RGC density than the PBS-treated group in the mid-peripheral retina (**d**) (544±66/mm^2^ *versus* 323±92/mm^2^, respectively). **P⩽0.01, ***P⩽0.001; *n*=6

To validate the FVEP outcomes, we performed retrograde tracing of RGCs to calculate the RGC density at day 28 post-infarct. The RGC densities of the sham, PBS-, 2 *μ*g P-sel-, and 4 *μ*g P-sel-treated groups in the central retina were 1841±139, 612±31, 825±365, and 1009±177 cells/mm^2^, respectively. The RGC densities of the sham, PBS-, 2 *μ*g P-sel-, and 4 *μ*g P-sel-treated groups in the mid-peripheral retina were 1063±92, 323±93, 544±66, and 614±99 cells/mm^2^, respectively. The survival rates of RGCs in the central retina were 33.2%, 44.8%, and 54.8% in the PBS-, 2 *μ*g P-sel-, and 4 *μ*g P-sel-treated groups, respectively. The survival rates of RGCs in the mid-peripheral retina were 30.5%, 51.1%, and 57.7% in the PBS-, 2 *μ*g P-sel-, and 4 *μ*g P-sel-treated groups, respectively. There was a significant increase in RGC density between the 4 *μ*g P-sel- and PBS-treated groups in both the central (Figures 2a and d; *P*=0.002) and mid-peripheral (Figures 2b and c; *P*=0.006) retina. However, the RGC density in the 2 *μ*g P-sel-treated group was significantly increased only in the mid-peripheral retina (Figure 2d; *P*=0.009), suggesting a dose-dependent effect. Together, these results validate our FVEP data and show that P-sel-IgG treatment increases the survival rate of RGCs in a dose-dependent manner.

### P-sel treatment rescues RGCs from apoptosis

**Figure 3:**
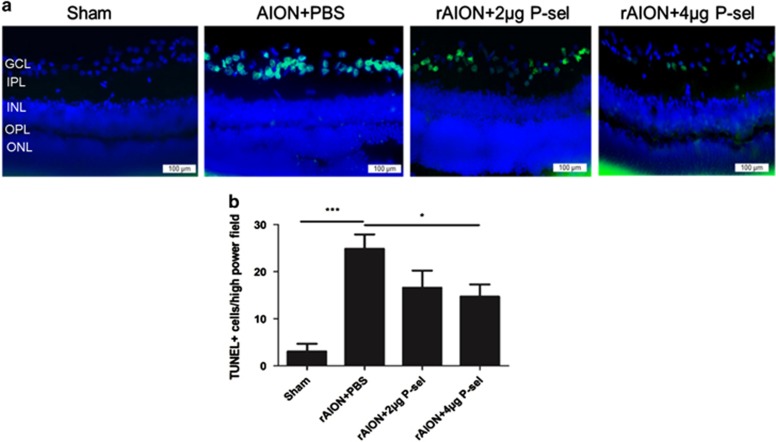
TUNEL assay in the retina. (**a**) Representative images of TUNEL-stained retinal cross sections after rAION in each group. (**b**) The 4 *μ*g P-sel-treated group showed significantly fewer TUNEL+ cells than the PBS-treated group in the central retina (13.30±6.290717706 *versus* 24.5±8.06, respectively). GCL, ganglion cell layer; IPL, inner plexiform layer; INL, inner nuclear layer; OPL, outer plexiform layer; ONL, outer nuclear layer; *P⩽0.05, ***P⩽0.001; *n*=6

To check whether P-sel-IgG can rescue RGCs from apoptosis, we performed an *in situ* TUNEL assay on retinal cross-sections. The numbers of TUNEL^+^ cells in the sham, PBS-, 2 *μ*g P-sel-, and 4 *μ*g P-sel-treated groups were 3±2, 24±8, 16±4, and 13±6, respectively. There was a significant reduction in TUNEL^+^ cells in the 4 *μ*g P-sel-treated group compared with the number in the PBS-treated group, but there was no significant difference between the PBS- and 2 *μ*g P-sel-treated groups (Figures 3a and b; *P*=0.01), further suggesting a dose-dependent effect. This result shows that P-sel-IgG treatment can rescue RGCs from undergoing apoptosis.

### P-sel prevents blood-borne macrophage infiltration in ON tissue

**Figure 4:**
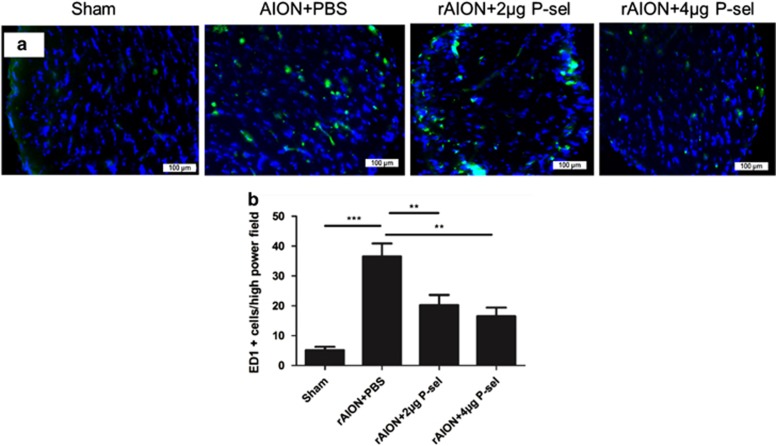
ED1 immunostaining of the ON. (**a**) Representative images of ED1 immunostaining in ON cross-sections after rAION in each group. (**b**) The 4 *μ*g P-sel- and 2 *μ*g P-sel-treated groups showed significantly fewer ED1+ cells than the PBS-treated group (16.53±10.26 and 20.2±10.29 *versus* 36.5±11.3, respectively). **P⩽0.01, ***P⩽0.001; *n*=6

Blood-borne macrophage infiltration into ON tissue is considered a primary response to tissue inflammation after AION.^12^ Hence, we performed immunostaining for ED1 in ON tissue to determine whether P-sel treatment could reduce blood-borne macrophage infiltration. ED1 immunostaining was performed at day 28 post-infarct. The numbers of ED1-positive cells in the sham, PBS-, 2 *μ*g P-sel-, and 4 *μ*g P-sel-treated groups were 5±4, 36±11, 20±10, and 16±10, respectively. There was a significant reduction in ED1-positive cells in the 2 *μ*g P-sel- and 4 *μ*g P-sel-treated groups (Figures 4a and b; 2 *μ*g P-sel, *P*=0.008; 4 *μ*g P-sel, *P*=0.002). These results show that P-sel-IgG treatment can reduce blood-borne macrophage infiltration in rAION ON tissue.

### OCT reveals a reduction in ON edema and preserved retinal nerve fiber layer (RNFL) thickness by P-sel treatment

**Figure 5:**
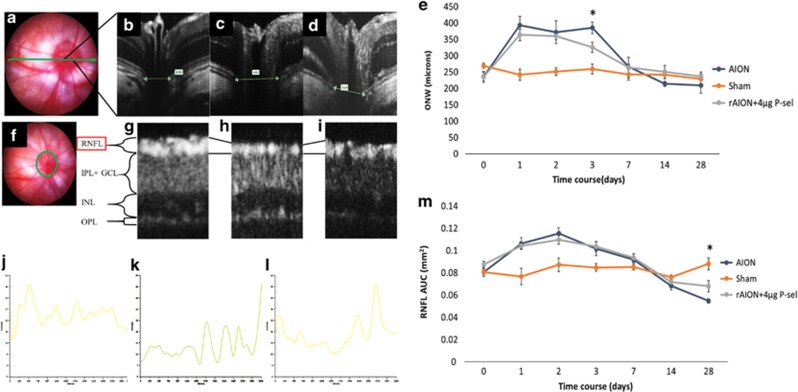
OCT profile of RNFL and ONW. (**a**) Linear scan across the optic nerve head. (**b**–**d**) Representative ONW profiles of the sham, rAION and 4 *μ*g P-sel-treated groups at day 3. (**e**) ONW thickness profile over time. Compared with the PBS-treated group, the 4 *μ*g P-sel-treated group exhibited a significant reduction in edema (385.25±48 *μ*m *versus* 325.5±37.3, respectively). (**f**) Circular scan around the optic nerve head. (**g**–**i**) Representative RNFL thickness measurement of the sham, rAION and 4 *μ*g P-sel-treated groups at day 28 (the black line indicates the RNFL). (**j**–**l**) Representative ONW profile of the sham, rAION and 4 *μ*g P-sel-treated groups at day 28. (**i**) RNFL thickness profile (area under the curve) over time. Compared with the PBS-treated group, the 4 *μ*g P-sel-treated group exhibited significant preservation of the RNFL at day 28 (0.5±0.15 mm^2^ *versus* 0.68±0.17 mm^2^, respectively). RNFL, retinal nerve fiber layer; GCL, ganglion cell layer; IPL, inner plexiform layer; INL, inner nuclear layer; OPL, outer plexiform layer; *P⩽0.05; *n*=6

In a previous report, we showed that the acute phase of rAION involved inflammation in ON tissue, possibly caused by a large amount of macrophage infiltration,^12^ which potentially caused ON edema in the acute phase. In our previous experiment, the 4 *μ*g P-sel-treated group showed more promising results and was thus chosen for further experiments. ON edema occurred immediately after AION induction; severe edema was observed at day 1 and completely recovered at day 7 (Figure 5e,Supplementary Table 3). We assumed that P-sel-IgG could reduce ON edema earlier in the course of rAION. We used spectral domain OCT to monitor optic nerve width (ONW) over time. There was a significant reduction in ON edema at day 3 in the 4 *μ*g P-sel-treated group (Figures 5b–e; *P*=0.041) compared with edema in the PBS-treated group. Additionally, we monitored RNFL thickness over time. We observed an increase in RNFL thickness until day 3 due to ON edema (Figure 5m,Supplementary Table 4). RNFL thickness in the chronic phase (day 14 and day 28) indicated that the change in thickness due to ON edema was completely reduced at day 7 in all groups with rAION. Hence, any changes in RNFL thickness after complete ON edema recovery was exclusively due to 4 *μ*g P-sel or PBS treatment. There was no significant reduction in ON edema in the 4 *μ*g P-sel-treated group. However, RNFL thickness was significantly preserved in the 4 *μ*g P-sel-treated group (Figures 5i; *P*=0.017) compared with RNFL thickness in the PBS-treated group at day 28. Together, these data suggest that P-sel-IgG can reduce edema in the acute phase and preserve RNFL thickness in the chronic phase.

### P-sel-IgG treatment stabilizes the BONB in the acute phase of rAION

**Figure 6:**
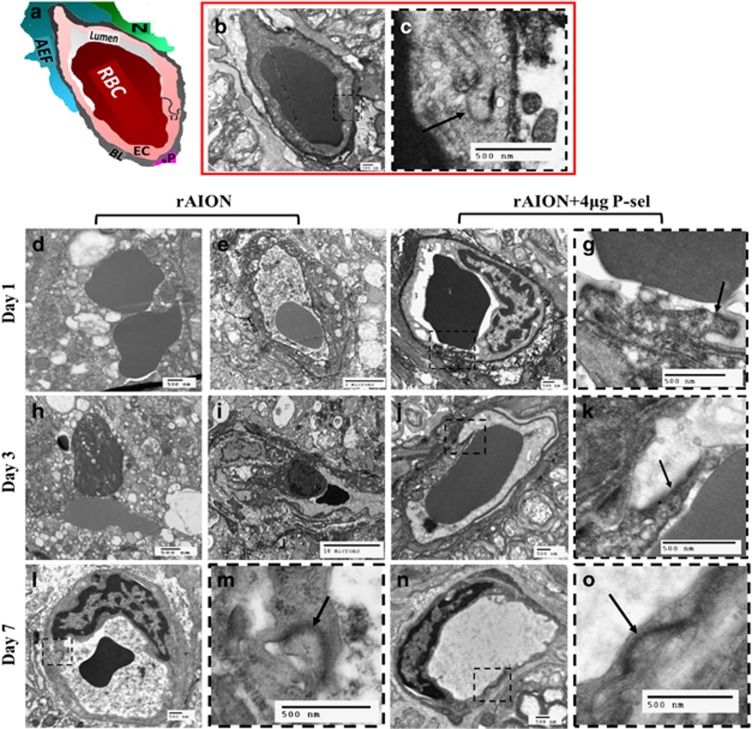
TEM of optic nerve cross sections. (**a**) Pictorial representation of the neurovascular unit with its major components (red blood cell, RBC (red); basal lamina, BL (dark gray); neurons, N (green); astrocyte end feet, AEF (blue); endothelial cell, EC (pink); pericyte, P (magenta)). (**b**) Cross-section image of a capillary of a sham. Intact ultrastructure with distinguishable components of the neurovascular unit; (*n*=1). (**c**) Inset with prominent tight junctions (black arrows). (**d,h**) Blood-optic nerve barrier (BONB) disruption with all components missing and (**e,i**) severe vacuolation in the BONB at day 1 and day 7 in the PBS-treated group; (*n*=2). (**f**, **j**) Preserved BONB with visible tight junctions (inset (**g,k**), black arrows) in the 4 *μ*g P-sel-treated group at day 1 and day 3; (*n*=2). (**l**) Reconstitution of the BONB at day 7 in the PBS-treated group. (**m**) Inset showing tight junctions; (*n*=1). (**n**) The BONB of the 4 *μ*g P-sel-treated group at day 7. (**o**) Inset showing tight junctions

rAION causes endothelial cell damage^1^ and increases vascular permeability.^32^ Therefore, we decided to perform transmission electron microscopy (TEM) to study changes in ON tissue. Based on our OCT results (Figure 5e), we limited our study to ultrastructural changes in the acute phase (until day 7). A sham ON was used to compare ON ultrastructure. All the ultrastructures of the capillaries were clearly visible (Figures 6b and c) in the sham ON. These capillaries in the ON act as the BONB. TEM revealed severe ultrastructural defects in the ONs of the PBS-treated group at day 1. The basal lamina was completely ruptured, and key components of the BONB were missing (Figure 6d). Most capillary units were completely damaged, but some exhibited compact basal lamina with severe vacuolation, endothelial cell damage (Figure 6 e) and missing tight junctions. Similar findings were observed at day 3 (Figures 6h and i), but the number of completely damaged capillaries was reduced, and capillaries with compacted basal lamina were observed more often, indicating the transition state in the reconstitution of the BONB. When we examined the 4 *μ*g P-sel-treated group, we observed dramatic protection from rAION injury. P-sel treatment stabilized the BONB, and the ultrastructure of the BONB was maintained at day 1 (Figure 6f) with observable tight junctions (Figure 6g). Although there was some endothelial cell damage at day 1, the tight junctions and basal lamina were still intact, and endothelial cell damage recovered at day 3. In addition, endothelial cells at day 7 in the 4 *μ*g P-sel-treated group (Figures 6n and o) closely resembled those in the sham group, whereas endothelial cell damage was present in the PBS-treated group (Figures 6i and m) at day 7. These findings account for the previous OCT results (Figure 5) in which ON edema was reduced in the 4 *μ*g P-sel-treated group in the acute phase. This result suggests that P-sel-IgG is protective by stabilizing the BONB in the acute phase of rAION.

### P-sel-IgG exhibits a Nrf2-mediated protective effect in the retina

**Figure 7:**
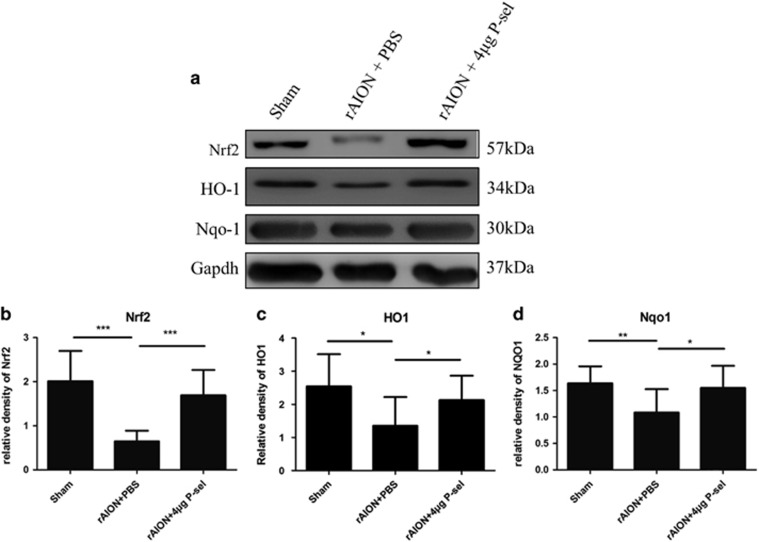
Immunoblots of the retina. (**a**) Representative cropped blot images of Nrf2, NQO1, and GAPDH (internal loading control). (**b,c**) Bar charts showing the relative density of Nrf2, HO-1 and Nqo1 with a sham retina as a reference. *P⩽0.05, **P⩽0.01***P⩽0.001; *n*=3

NRF2 is needed for PSGL-1-mediated protection of the liver following ischemia-reperfusion injury.^27^ PSGL-1 is a well-known ligand of P-selectin; therefore, we targeted Nrf2 and other AREs. Nrf2 expression significantly increased in the 4 *μ*g P-sel-treated group (Figure 7b) compared with expression in the PBS-treated group. The expression levels of two AREs (Nqo1 and Ho1) were also significantly increased in the 4 *μ*g-P-sel-treated group (Figure 7c). This result shows that P-sel-IgG exerts neuroprotection via the Nrf2 signaling pathway.

## Discussion

**Figure 8:**
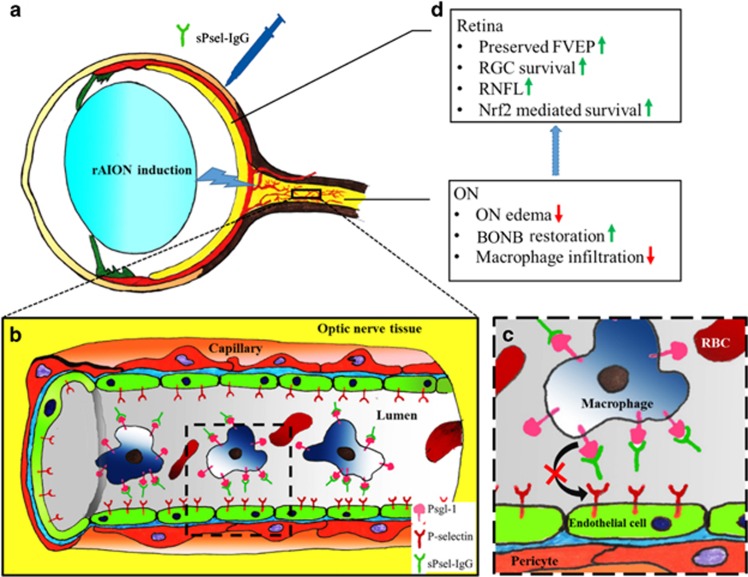
Summary of this study (**d**) and a hypothetical model for the neuroprotective effect of P-selectin-IgG in the rAION model. P-sel-IgG treatment after rAION induction (**a**) can saturate Psgl-1 (**b**, inset **c**) and stop macrophage infiltration in optic nerve tissue

Our observations demonstrate that P-sel-IgG administration at day 1 can rescue RGCs from apoptosis and preserve visual function by stabilizing the BONB in rAION model. The stabilized BONB (Figure 6) explains the reduction in ON edema at day 3 (acute phase) (Figure 5), which will prevent secondary RGC death in the chronic phase after rAION induction, as demonstrated by TUNEL staining (Figure 3), RGC density (Figure 2) and FVEP results (Figure 1) at day 28 post-infarct. BONB stabilization plays an important role in rescuing RGCs in the acute phase and maintaining the RGC population over time. This finding is consistent with previous reports that BONB stabilization can reduce inflammation and macrophage migration in ON tissue.^32^ Furthermore, we demonstrated that P-sel-IgG exerts neuroprotection via the Nrf2 signaling pathway, consistent with a previous study that used a similar kind of PSGL-1 recombinant protein.^27^ In addition, Nrf2 activation is protective in intracerebral hemorrhage^33^ and middle cerebral artery occlusion in the brain,^34^ which are also CNS disorders, along with AION. P-selectin is involved in maintaining blood–brain–barrier integrity^35^; in a similar manner, PSGL-1 inhibition stabilized the BONB in this study. Based on our results, we can assume that P-sel-IgG acts in a dose-dependent manner as RGC density was not significantly affected in the mid-peripheral region, and the TUNEL assay also showed no significant reduction in the 2 *μ*g P-sel-treated group whereas 4 *μ*g P-sel-treated group was effective significantly throughout all the experiments. One potential explanation is that an optimum concentration of P-sel-IgG is needed to saturate all Psgl-1 molecules to prevent endogenous P-selectin binding; however, this approach may be ineffective. When P-sel is applied to other disease models, this limitation and alternative administration routes should be considered. We assumed that P-sel-IgG would bind to Psgl-1 (Figure 8) because a previous study reported a strong interaction between them.^36^ PSGL-1 is also a ligand of P-, L- and E-type selectins, but it has the highest affinity for P-selectin.^37^ Hence, using P-sel-IgG was a more reliable approach to stop interactions between Psgl-1 and all three types of selectins (three targets in a single-hit approach), making it a better candidate than directly inhibiting P-selectin. This study is the first to use P-sel-IgG as a treatment for rAION.

P-selectin is used as a disease marker for cardiovascular disorder,^38^ rheumatoid arthritis, and arthritis^39^; furthermore, elevated levels of P-selectin are present in AION patients.^40^ The soluble form of P-selectin is the result of proteolytic cleavage of the extracellular domain after interacting with PSGL-1. This cleavage is a defense mechanism that downregulates the inflammatory response of platelets. P-selectin may have to dimerize to strongly bind to PSGL-1, and the monomeric form of P-selectin has less affinity for Psgl-1,^36^ which may explain why soluble p-selectin in the blood only indicates disease risk, not the disease itself. We used the dimeric form of P-sel-IgG, which may be more effective.

High concentrations of the dimeric form of P-selectin can cause coagulation in mice,^36^ and several clinical trials conducted using P-selectin antagonists were also stopped due to its severe side effects.^41^ Recently, a human study of inclacumab, a monoclonal antibody against P-selectin, was conducted to address issues of tolerability, pharmacokinetics, and pharmacodynamics in healthy controls before using it on patients. This study showed no adverse effects of inclacumab.^42^ Therefore, we used an intravitreal injection (IVI) to limit the spread of P-sel-IgG to the injury site, which reduces the risk of adverse effects in other parts of the body.

The stress-sensing transcription factor Nrf2 is the key regulator for the expression of a majority of endogenous antioxidant enzymes. Under physiological redox status, Nrf2 undergoes proteosomal degradation by Keap1/Nrf2 complex.^34^ Upon oxidative stress, Nrf2 is released from Keap1, translocated into the nucleus, and activates the transcription of target genes, such as superoxides dismutases, catalase, glutathione peroxidases, peroxiredoxins, heme oxygenases and Nqo-1.^43, 44^ As shown in this study, AION induction significantly reduced Nrf2 levels and downregulated the antioxidant enzymes, HO-1 and Nqo1. P-sel-IgG treatment can activate Nrf2 expression and significantly upregulate the expression level of Nqo1 and HO-1. Targeting Nrf2-ARE pathway may be a potential therapeutic approach as its activation was found neuroprotective in retinal ischemic injury,^45, 46^ intracerebral hemorrhage,^33^ and middle cerebral artery occlusion in brain.^34^ Despite several studies involving Nrf2 pathway in various ischemic diseases, to the best of our knowledge, our study is the first to demonstrate the contribution of the Nrf2 antioxidant pathway in rAION model.

In conclusion, this study provides insights into the neuroprotective effects of P-sel-IgG in a rAION model (Figure 8). Our novel findings suggest that P-sel-IgG protects RGCs by stabilizing the BONB and activating the Nrf2-ARE signaling pathway. P-sel-IgG would be a potential therapeutic application for the treatment of ischemic ON and retinal vascular diseases. Since the ON is part of the CNS, and AION pathology is similar to other types of stoke in the CNS, P-sel-IgG treatment may also be effective for treatment of other types of CNS strokes or white matter ischemia.

## Materials and Methods

A list of resources used in this study is provided in Supplementary Table 1.

### Animals

Sixty-one outbred adult Wistar rats weighing 150–180 grams (7–8 weeks) were maintained in filter top holding cages. The rats had free access to food and water in an environmentally controlled room at a temperature of 23 °C and 55% humidity with a 12-h light-dark cycle (light period 7 a.m.–p.m.). Animal care and experimental procedures were conducted in accordance with the ARVO statement for the use of Animals in Ophthalmic and Vision Research, and the Institutional Animal Care and Use Committee (IACUC) at the laboratory animal center, Tzu Chi University approved all the animal experiments. An intramuscular injection of a ketamine (100 mg/kg) and xylazine (10 mg/kg) cocktail was administered for general anesthesia. Alcaine was applied for local anesthesia, and Mydrin-P was applied for pupil dilation in all the experiments. Study design details are provided in Supplementary Table 2.

### AION induction

Alcaine and Mydrin-P eye drops were applied for local anesthesia and pupil dilation, respectively. After general anesthesia, 2.5 mM rose bengal in PBS (1 ml/kg animal weight) was intravenously administered. Immediately after rose bengal injection, the optic disc was exposed to an argon green laser (532 nm wavelength, 500 mm size and 80 mW power) for 12 1-s pulses. A fundus lens was used to focus the laser on the optic disc. Tobradex eye ointment was applied after the procedure, and the rats were monitored until complete recovery was observed.

### P-sel-IgG administration and formulation

We used recombinant mouse P-selectin-Fc chimera protein (P-sel-IgG), which comprises a C-type lectin domain and an EGF-like domain of P-selectin fused with the Fc region of human IgG_1_ in a disulfide-linked homodimer form. In brief, 200 *μ*g P-sel-IgG was reconstituted in a 200 *μ*l PBS:glycerol (8 : 2) solution to achieve a 1 *μ*g/*μ*l concentration. The animals were either treated with PBS, 4 *μ*g P-sel-IgG (4 *μ*g P-sel), or 2 *μ*g P-sel-IgG (2 *μ*g P-sel) in a total volume of 4 *μ*l by IVI.

### Flash visually evoked potential recordings

After general anesthesia, the sagittal region of the skull was opened. Screw implants were fixed at the primary visual cortex region of both hemispheres using stereotaxic coordinates (AP: anterior-posterior; ML: medial-lateral; DV: dorsal-ventral; AP: −8 mm; and ML: −3.0 mm); one electrode was fixed at the frontal cortex (AP: 3 mm). FVEPs were measured using a visual electrodiagnostic system. The system has built-in programs to measure FVEPs. Electrodes at the primary visual cortex were considered active (positive) electrodes, the electrode at the frontal cortex was considered the reference (negative) electrode, and the ground electrode was placed in the rat’s tail. The settings used were as follows: no background Illumination, a flash intensity of 30 cd.s/m^2^, and a single flash with a flash rate of 1.02 Hz. An average of 64 sweeps were collected, and the raw data were saved for further analysis. The P1-N2 amplitude was measured to check visual function.

### Retrograde labeling of RGCs by fluoro-gold and measurement of RGC density

RGCs were labeled in a retrograde manner as described in our previous report.^47^ In brief, retrograde labeling was performed 1 week before the rats were sacrificed. The sagittal region of the skull was opened, and 2 *μ*l fluoro-gold was injected into the superior colliculus (AP: −6 mm; ML: −1.5 mm; and DV 4 mm). The same procedure was performed on the other hemisphere. One week after labeling, the rats were killed, and the eyeballs were collected and fixed in 10% formalin. Retinas were carefully flat mounted. The retina was examined under a fluorescence microscope with × 100 power, an inbuilt filter set (excitation filter, 350–400 nm; barrier filter, 515 nm) and a connected digital imaging system. The retina was examined from 1 mm to 3 mm from the center to calculate central and peripheral RGC densities. At least 10 random regions were separately scanned in the central and mid-peripheral regions; images of these cells were saved for density calculation. RGC density was calculated by ImageMaster 2D Platinum software. The RGC survival rate was determined by calculating the ratio of the treatment groups to the sham-operated group and multiplying the ratio by 100.

### Retinal and ON sample preparation

The rats were killed, and their eyes were enucleated and fixed in 4% paraformaldehyde. The eyeballs and ONs were separated and transferred to 30% sucrose; the samples were stored at 4 °C until they settled at the bottom of the tubes. Retina and ON cross sections of 20 *μ*m were obtained using a cryostat.

### ED-1 immunohistochemistry (IHC) on ON tissues

Anti-ED-1 is specific for extrinsic macrophages. ON cross-sections were blocked with 5% FBS for 1 h at room temperature. The tissue was labeled with an ED1 primary antibody diluted in antibody dilution buffer (2% BSA, 1 × PBS (pH 7.2), and 0.3% Triton X-100; 1 : 200) overnight at 4 °C. Goat anti-mouse Alexa 488 (0.3% Triton X-100 and 1 × PBS (pH 7.2); 1 : 500) was added to the tissues, which were incubated for 1 h at room temperature and counterstained with DAPI (0.3% Triton X-100 and 1 × PBS (pH 7.2); 1 : 500). Image acquisition was conducted with appropriate filter sets in a fluorescence microscope at × 100 magnification. ED-1^+^ cell counting was manually performed or conducted by ImageMaster 2 Platinum software.

### TUNEL assay

TUNEL was used to detect apoptotic cells in the ganglion cell layer (GCL). A TUNEL assay was performed according to the manufacturer’s protocol (DeadEnd Fluorometric TUNEL System; Promega Corporation, Madison, WI, USA). TUNEL^+^ cells in the GCL were manually counted.

### Image-guided OCT imaging

A Phoenix Micron IV retinal microscope with image-guided OCT was used for imaging. This system uses spectral domain OCT, which provides a longitudinal resolution of 1.8 *μ*m and a transverse resolution of 3 *μ*m with a 3.2-mm field of view and 1.2-mm imaging depth at the retina. After general anesthesia, the rats were placed on the imaging platform, and the head was positioned at an angle to allow the penetration of light vertical to the cornea from the temporal side. The RNFL was obtained by circular scanning around the optic disc, and the Bruch membrane opening (ONW) was scanned by a linear scan through the center of the optic disc. At least three clear captures were obtained for each eye. Quantitative measurements of the Bruch membrane opening and RNFL thickness were carried out by built-in ‘Insight’ software. This software generates a segment of different layers and a thickness profile of the desired segmented layer. The average RNFL thickness was measured by calculating the area under the curve for the RNFL thickness profile with GraphPad Prism. The above-mentioned procedure was performed at pre-rAION (day 0) and at day 1, day 3, day 7, day 14 and day 28 post-rAION.

### Transmission electron microscopy of ON

The rats were killed at different time points (day 1, day 3, and day 7), and the ON tissues (1 to 2 mm^3^) were dissected 1 mm away from the ON head. The tissues were prefixed in 2.5% glutaraldehyde/0.1 M cacodylate buffer + 1% tannic acid. The tissues were then post-fixed with 1% osmium tetroxide/0.1 M cacodylate buffer. After post-fixation, the tissues were subjected to en block staining with 2% uranyl acetate. The tissues were then embedded in Spurr’s resin, and 80-nm-thick cross-sections were obtained with an ultra-microtome and observed by TEM. An average of 4–5 microphotographs of capillaries were taken per sample at the desired magnification.

### Western blotting

The rats were killed, and their eyes were enucleated. The retinas were homogenized and stored at −80 °C for further analysis. A protein assay was performed using a BCA protein assay kit. For immunoblotting, 30 *μ*g of protein was separated on a 10% bis-acrylamide gel. The proteins were transferred to polyvinylidene difluoride membranes. After the transfer, the membranes were blocked with 5% non-fat dry milk for 1 h, followed by an overnight incubation with Nrf2 (1 : 250; Santa Cruz Biotechnology, Santa Cruz, CA, USA), Nqo1 (1 : 500; Santa Cruz), Ho1 (1 : 1000; Abcam, Cambridge, MA, USA), or GAPDH (1 : 2000; Sigma-Aldrich, St. Louis, MO, USA) primary antibody at 4 °C. The membranes were washed, followed by incubating with a secondary antibody conjugated to HRP against the appropriate host species for 1 h at room temperature. The membranes were then developed using enhanced chemiluminescent substrate, and images were taken in a western blot analyzer. The relative density was calculated using ImageJ software.

### Statistical analysis

All statistical analyses was performed using GraphPad Prism. The data are presented as the mean±S.D. A Mann–Whitney *U*-test was used for comparisons between groups. *P*-values less than 0.05 were considered statistically significant, with * representing *P*⩽0.05, **P⩽0.01, and ***P⩽0.001.

## Publisher’s Note

Springer Nature remains neutral with regard to jurisdictional claims in published maps and institutional affiliations.
