# Supplementary material for: Soluble P-selectin promotes retinal ganglion cell survival through activation of Nrf2 signaling after ischemia injury
Source: Cell Death Dis. 2017 Nov 16;8(11):e3172–. doi: 10.1038/cddis.2017.566 (PMC5775414; doi:10.1038/cddis.2017.566)
Supplement: Supplementary Information [file cddis2017566x1.doc]

**Supplementary information**

**Soluble P-selectin promotes retinal ganglion cell survival through activation of Nrf2 signaling after ischemia injury**

Kishan Kapupara^1^, Yao-Tseng Wen^2^, Rong-Kung Tsai^2,3^, and Shun-Ping Huang^1 *^

^1^ Department of Molecular Biology and Human Genetics, Tzu-Chi University, Hualien 970, Taiwan.

^2^ Institute of Eye Research, Buddhist Tzu Chi General Hospital, Hualien, Taiwan

^3^ Institute of Medical Sciences, Tzu Chi University, Hualien, Taiwan

**Correspondence:**

**Shun-Ping Huang**

**Department of Molecular Biology and Human Genetics,**

**Tzu Chi University**

**701, Sec 3, Chung-Yang Rd, Hualien 97002, Taiwan.**

**Tel: +886-3-8565301 ext 2664**

**Fax: +886-3-8561422**

**E-mail:** [**sphophdoc1688@gmst.tcu.edu.tw**](mailto:sphophdoc1688@gmst.tcu.edu.tw)**, sphophdoc1688@gmail.com**

**Materials and methods**

**Table 1 List of resources used in this study**

| **Reagent or resource** | **Source** | **Identifier** |
| --- | --- | --- |
| **Antibodies and recombinant proteins** | | |
| Goat anti-mouse Alexa 488 | Life Technologies OR, USA | Cat#A11001 Lot# 1613346 |
| Goat anti-mouse HRP | Bio-Rad Laboratories, Inc., CA, USA | Cat#170-6516 |
| Goat anti-rabbit HRP | Jackson Immuno research laboratories, Inc., PA, USA | Cat#111-035-00, Lot# 126526 |
| Mouse monoclonal anti-CD68 | Bio-Rad Laboratories, Inc., CA, USA | Cat#MCA341GA |
| Mouse monoclonal anti-GAPDH | Sigma-Aldrich co., MO, USA | CAT# G8795 |
| Mouse monoclonal anti-NQO1 | Santa Cruz Biotechnology, Inc., USA | Cat# sc-32793, Lot# K2816 |
| Rabbit polyclonal anti-HO-1 | Abcam, MA, USA | Ca#-ab13243 |
| Rabbit polyclonal anti-Nrf2 | Santa Cruz Biotechnology, Inc., USA | Cat# sc-722, Lot# I2211 |
| Recombinant Mouse P-Selectin/CD62P Fc Chimera Protein | R&D Systems, Inc. MN | Cat# 737-PS, Lot# DIF0814121 |
| **Commercial assays** | | |
| Protein BCA kit | Thermo Scientific, IL, USA | Cat# 23225 Lot# OA183168 |
| TUNEL assay | Promega Corporation, WI, USA | Cat#G3250, Lot#0000180289 |
| **Animal model** | | |
| Outbred male Wistar rats | BioLASCO Taiwan Co., Ltd., Taiwan | N/A |
| **Equipment** | | |
| Chemiluminescence Western blot imaging | UVP, LLC, CA, USA | Cat# BioSpectrum 810, N/A |
| Cryostat (cryosectioning) | Leica Microsystems, Germany | Cat# Leica CM3050S, N/A |
| Fluorescence microscope | Carl Zeiss Meditech Inc., Thornwood, NY, USA | Cat# Axioplan 2 imaging, N/A |
| FVEP stimulator | Diagnosys LLC, MA, USA | Cat# Colordome ganzfeld, N/A |
| Green Laser Photocoagulator | NIDEK CO., LTD, Japan | Cat# GYC-500, N/A |
| Spectral domain OCT | Phoenix research labs, CA, USA | Cat# Micron IV, N/A |
| Transmission electron microscope | Hitachi High-Technologies Corporation, japan | Cat# Hitachi H-7500, N/A |
| Ultramicrotome | Leica Microsystems, Germany | Cat# Leica EM UC6, N/A |
| **CHEMICALS pharmaceutical grade** | | |
| Balanzine(Xylazine 2%w/v) | Health-Tech Pharmaceutical Co., Taipei, Taiwan | Cat# Balanzine 2%, Lot# 502001 |
| Fluoro-gold | Flurochrome LLC, Denver, CO, USA) | N/A |
| Imalgene 1000(ketamine 100mg/ml) | Merial, France | Cat# Imalgene 1000, Lot# LBM155AA |
| Phenylephrine hydrochloride eye drops | Santen Pharmaceutical, Osaka, Japan | Cat# Mydrin-P, Lot#mp2010 |
| Proparacaine Hydrochloride Ophthalmic Solution | Alcon-Couvreur, N.V., Puurs, Belgium | Cat# Alcaine, Lot#16e26ed |
| Rose bengal | Sigma-Aldrich Co., MO, USA | Cat# R4507, N/A |
| Tobramycin, Dexamethasone | Alcon-Couvreur, N.V., Puurs, Belgium | Cat# Tobradex, Lot# 13J30K |
| **Reagents, Buffers, and solutions** | | |
| Bis-acrylaminde | Bio-Rad Laboratories, Inc., CA, USA | Cat# 161-0156, N/A |
| FBS | Gibco life technologies, USA | Cat#26140-079 |
| Glutaraldehyde | Electron microscopy sciences, PA, USA | Cat# 16220, N/A |
| Immobilon-P^SQ^(PVDF membrane) | Millipore corporation, MA, USA | Cat# ISEQ00010, Lot# K2MA7796H |
| Methanol | Avantor performance materials. Inc. PA, USA | Cat# 9093-68, Lot# 0000067375 |
| Osmium tetroxide | Electron microscopy Sciences, PA, USA | Cat# 19190, N/A |
| PBS | Gibco life technologies, USA | Cat#70011-044 |
| Sodium cacodylate | Electron microscopy sciences, PA, USA | Cat# 12300, N/A |
| Spurr’s resin | Electron microscopy sciences, PA, USA | Cat# 14300, N/A |
| Uranyl acetate | Electron microscopy sciences, PA, USA | N/A, N/A |
| **Software and algorithms** | | |
| AMT camera system | Advanced Microscopy Techniques, Corp., MA, USA | N/A |
| Axiovision LE | Carl Zeiss micro imaging | N/A |
| Discover OCT | Phoenix research labs, CA, USA | N/A |
| Espion V6 | Diagnosys LLC, MA, USA | N/A |
| Image j | https://imagej.nih.gov/ij/ | N/A |
| Image Master 2D Platinum | GE Healthcare Bio-Sciences, Sweden | N/A |
| Insight | Phoenix research labs, CA, USA | N/A |

**Table 2 Summary of rats used in this study**

| **Experiments** | **Sham** | **rAION** | **rAION+2µg P-sel** | **rAION+4µg P-sel** |
| --- | --- | --- | --- | --- |
| FG/OCT | 6 | 6 | 6 | 6 |
| VEP/OCT/Immunoblot/IHC/TUNEL | 6 | 6 | 6 | 6 |
| TEM | 1 | 6 | 0 | 6 |
| **Total** | **61** | | | |

**Results**

**Table 3 ONW in time course. Data represented as meand ± SD; unit micron; n=6 (refer to figure 5 in main text)**

| **Time course** | **Sham** | **rAION+ PBS** | **rAION+4µg P-sel** | **p value** |
| --- | --- | --- | --- | --- |
| Day0 (pre-rAION) | 269.5±19.0 | 236± 40.3 | 235± 40.6 | n.s |
| Day 1 | 242.16± 40 | 393.6± 71.2 | 363.5± 43.8 | n.s |
| Day 2 | 251.16± 28.5 | 371.8± 94.7 | 360.33± 28.1 | n.s |
| Day 3 | 259.5± 37.6 | 385.25± 43.2 | 325.5± 37.4 | 0.041 |
| Day 7 | 242.8± 37.4 | 266± 21.7 | 263.83± 74.4 | n.s |
| Day 14 | 241.4± 24.7 | 214.25± 19.9 | 250.17± 50.7 | n.s |
| Day 28 | 229.16± 38.8 | 209.75± 61.59 | 237.33± 30.1 | n.s |

**Table 4 Time course data for RNFL thickness in time course. Data represented as mean ± SD; unit mm^2^; n=6 (refer to figure 5 in main text)**

| **Time course** | **Sham** | **rAION+ PBS** | **rAION+4µg P-sel** | **p value** |
| --- | --- | --- | --- | --- |
| Day0 (pre-rAION) | 0.080±0.009 | 0.082±0.008 | 0.087±0.009 | n.s |
| Day 1 | 0.0763±0.002 | 0.106±0.015 | 0.104±0.084 | n.s |
| Day 2 | 0.087±0.013 | 0.12±0.015 | 0.11±0.013 | n.s |
| Day 3 | 0.0848±0.0076 | 0.096±0.0189 | 0.108 ±0.014 | n.s |
| Day 7 | 0.0865±0.006 | 0.092± 0.009 | 0.0934± 0.0101 | n.s |
| Day 14 | 0.0759± 0.005 | 0.0684±0.008 | 0.0759± 0.00550 | n.s |
| Day 28 | 0.0857± 0.0122 | 0.0547± 0.00497 | 0.0679± 0.0174 | 0.0175 |
